# Real-Time Hyperspectral Video Acquisition with Coded Slits

**DOI:** 10.3390/s22030822

**Published:** 2022-01-21

**Authors:** Guoliang Tang, Zi Wang, Shijie Liu, Chunlai Li, Jianyu Wang

**Affiliations:** 1 Key Laboratory of Space Active Opto-Electronics Technology, Shanghai Institute of Technical Physics, Chinese Academy of Sciences, Shanghai 200083, China; tangguoliang@mail.sitp.ac.cn (G.T.); wangzi@shanghaitech.edu.cn (Z.W.); 2 University of Chinese Academy of Sciences, Beijing 100049, China; 3 Hangzhou Institute for Advanced Study, University of Chinese Academy of Sciences, Hangzhou 310024, China; liushijie@ucas.ac.cn

**Keywords:** computational imaging, video spectral imaging, aperture coded

## Abstract

We propose a real-time hyperspectral video acquisition system that uses coded slits. Conventional imaging spectrometers usually have scanning mechanisms that reduce the temporal resolution or sacrifice the spatial resolution to acquire spectral information instantly. Recently, computational spectral imaging has been applied to realize high-speed or high-performance spectral imaging. However, the most current computational spectral imaging systems take a long time to reconstruct spectral data cubes from limited measurements, which limits real-time hyperspectral video acquisition. In this work, we propose a new computational spectral imaging system. We substitute the slit in a conventional scanning-based imaging spectrometer with coded slits, which can achieve the parallel acquisition of spectral data and thus an imaging speed that is several times higher. We also apply an electronically controlled translation stage to use different codes at each exposure level. The larger amount of data allows for fast reconstruction through matrix inversion. To solve the problem of a trade-off between imaging speed and image quality in high-speed spectral imaging, we analyze the noise in the system. The severe readout noise in our system is suppressed with S-matrix coding. Finally, we build a practical prototype that can acquire hyperspectral video with a high spatial resolution and a high signal-to-noise ratio at 5 Hz in real time.

## 1. Introduction

Imaging spectrometers acquire two-dimensional (2D) spatial and one-dimensional (1D) spectral information from a scene simultaneously, producing three-dimensional (3D) data cubes. Since different materials have different spectral characteristics, obtaining 3D spectral images of scenes is critical for activities such as material recognition, target detection, and remote sensing mapping.

It is inherently difficult to acquire a 3D data cube directly from a 2D detector array. Therefore, most spectral imaging approaches reconstruct 3D spectral data cubes from a projection of a 3D cube. According to the means used for projection, we can classify spectral imaging techniques as either scanning, direct imaging, or computational imaging.

One way to achieve scanning-based spectral imaging is to mount a panchromatic camera with a controllable bandpass filter, such as an acousto-optic tunable filter (AOTF) [1] or a liquid crystal tunable filter (LCTF) [2]. Each measurement produces a grayscale image at one wavelength, and all images across different wavelengths are stacked to generate a 3D data cube. In addition to spectral scanning, one-dimensional spatial scanning is also able to realize spectral imaging [3]. Such schemes adopt a single slit to limit the field-of-view of the imaging system, meaning that the 1D spatial and spectral information from the scene is collected in each measurement. The acquisition of a 3D data cube requires either the movement of the slit or the whole imaging system. Such scanning-based imaging spectrometers usually cannot realize real-time spectral video acquisition. Moreover, the noise in the system also requires the scanning-based imaging spectrometer to trade between imaging speed and image quality. High-speed imaging needs a short integration time and causes a weak light intensity, leading to the deterioration of the signal-to-noise ratio (SNR), and vice versa.

Direct imaging spectrometry uses 3D data cubes that can be generated by synthesizing measurement data in a simple way. The most direct imaging spectrometers sacrifice spatial resolution to capture light at different wavelengths. The earlier integral field spectrometer (IFS) adopts various optical elements, such as lenslets [4], fibers [5], and mirrors [6], to down-sample the 3D data cube in terms of spatial dimension. The spectrum is then dispersed and captured a using focal plane array (FPA), designed by shanghai institute of technical physics, for example ). IFS can obtain a spectral image at each measurement. However, the spatial and spectral resolution is limited due to the complex optical components and structure. Instead of a sophisticated optical design, the prism-mask spectral video imaging system (PMVIS) employs an occluding mask to achieve the controllable sampling of the scene and to realize a higher spatial and spectral resolution [7]. Recently, pixel-level filters have been developed to directly filter each pixel on the detector to capture spectral images [8]. Direct imaging spectrometers are able to achieve real-time imaging and video acquisition. However, they are still limited by the trade-off between the imaging speed and the SNR. Furthermore, the spatial resolution is sacrificed for spectral information in direct spectral imagers, limiting their applications in many areas.

Computational spectral imaging techniques provide new ideas to address the problem of the trade-off between spatial resolution, spectral resolution, and temporal resolution in spectral video acquisition. Most computational spectral imaging methods apply a more sophisticated approach to project the 3D data cube onto the 2D detector and reconstruct the 3D spectral image using various algorithms. For example, computed tomographic imaging spectrometry (CTIS) acquires the projections of 3D spectral data cubes in different directions and angles by dispersive devices, such as gratings, and reconstructs the spectral images with a filtered inverse projection [9] or iterative reconstruction algorithm [10]. However, CTIS only captures a small number of spectral data projections, leaving an intractable reconstruction problem. Therefore, the reconstruction results of CTIS are of low quality. At each exposure level, the coded aperture snapshot spectral imager (CASSI) measures the coded summation of the 3D data cube across the spectral dimension [11] or displaced spectral dimension [12]. In order to solve the problem of poor reconstruction quality owing to extremely limited information, various algorithms and upgraded settings, such as low rank [13], Gaussian mixture model [14], deep learning [15,16], and multi-frame CASSI [17], have been proposed to improve the reconstruction performance. For video-frame spectral imaging, various hybrid imaging systems that involve adding a high-speed RGB or monochromatic camera into current spectral imaging systems with data fusion algorithms have been proposed [18,19]. However, these systems usually require extensive computation time or rely upon large amounts of training data, which are difficult to achieve in practical spectral video acquisition systems.

In this paper, we propose a real-time hyperspectral video acquisition system (RHVS) based on coded slits. Our system can be categorized as a computational spectral imaging method. To accelerate the reconstruction process, we designed and assembled the full-rank imaging matrix using coded slits. The designed full-rank S-matrix enables real-time spectral video acquisition by solving the linear reconstruction problem in an analytical way. RHVS is different from the conventional scanning-based spectral imaging system in that the single slit is replaced with moveable coded slits. The coded slits enlarge the throughput of the system, suppressing the read noise in the detector and thus enabling a higher imaging speed in our proposed system. Compared to direct spectral imaging techniques, our method features a lossless spatial resolution. As a result, our proposed system can yield real-time hyperspectral video acquisition with a high SNR and spatial resolution.

Our contributions can be summarized as follows:

1. We propose a real-time hyperspectral video acquisition system that is realized by designed coded slits.
2. We analyze the noise in our proposed system and apply coded slits to suppress the read noise and realize high-speed imaging.
3. We build a practical prototype of proposed system and verified that the system achieves high temporal resolution, high spatial resolution, and a high signal-to-noise ratio.

In Section 2, we introduce the imaging and noise suppression principle of the proposed system. In Section 3, we describe the system design, implementation, and calibration in detail. In Section 4, we verify the effectiveness of the proposed system through synthetic and experimental results. In Section 5, we summarize our work and discuss the directions for future work.

## 2. Principles

### 2.1. Hyperspectral Video Acquisition System

**Figure 1:**
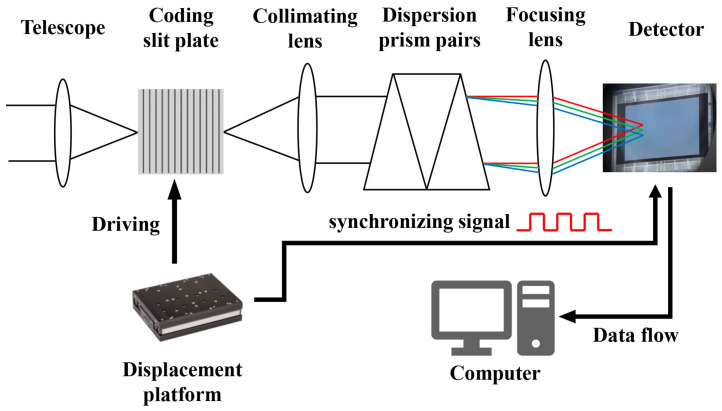
System schematic for the RHVS video acquisition.

Figure 1 shows the schematic of the proposed system. The incident light enters the system through the telescope and is modulated by the coded slits. The encoded light is dispersed by prism pairs and projected onto the detector through multiplexing. The coded slits can be translated parallel to the dispersion by a translation stage, thus changing the coding at each exposure. Since the imaging process for each row of the 3D spectral data cube is independent, we only analyze the signal flow in one row of the data cube.

**Figure 2:**
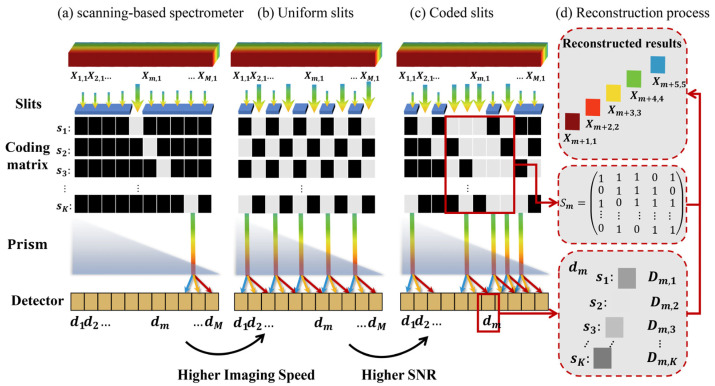
The imaging process of various hyperspectral imaging systems. The system with uniform slits exhibits a higher imaging speed compared with a scanning-based spectrometer. Additionally, the system with coded slits exhibits a higher SNR with respect to the system with uniform slits, as is illustrated in Section 2.2. In the reconstruction process of a system with coded slits, we extract the measurement matrix $S_{m}$ from coding matrix S and apply matrix inversion to the measurements to reconstruct a spatially shifted spectral vector. The details of this process are given in Section 2.1.

Our system principle is shown in Figure 2c,d. We denote $X_{m,n}$ as the light intensity of the n-th band at the m-th spatial location of the scene, where m=1,2,…,M, n=1,…,N, M denotes the length of the 3D data cube in one spatial dimension, and N is the number of bands. In the practical system, we consider M≫N. We also denote K as the exposure time for one spectral data cube, with $D_{m,k}$ being the measurement of the m-th pixel on the detector at the *k*-th exposure. We denote $s_{k}\in R^{M}$ as the coding at the *k*-th exposure, where $s_{k}$ is a one-dimensional vector consisting of only elements “0” and “1”. The element “1” represents the light transmission at the corresponding spatial location and “0” represents the occlusion. We use the translation stage to move the coded slits and change the coding, meaning that the (k+1)-th coding $s_{k+1}$ is a shifted result of the k-th coding $s_{k}$. Therefore, the coding of all K-times measurements forms a cyclic coding matrix $S={\left[s_{1},\ldots ,...,s_{K}\;\right]}^{T}$. The prism pairs project the light at the m-th spatial location of the spectral data cube to different positions on the detector according to the wavelength. Thus, the measurement on the m-th pixel on the detector is a superposition of the light intensity at different (m+i)-th spatial positions corresponding to the i-th band. Then, the measurement of m-th pixel of the detector at the k-th exposure is:

(1) $$D_{m,k}=\sum_{i=0}^{N}S_{k,m+i}X_{m+i,i},$$

We combine all K-times measurements of the m-th pixels and form a matrix multiplication:

(2) $$\left[\begin{array}{c} D_{m,1}\\ D_{m,2}\\ \ldots \\ D_{m,K} \end{array}\right]=\left[\begin{array}{c} S_{1,m+1,}S_{1,m+2},\ldots ,S_{1,m+N}\\ S_{2,m+1},S_{1,m+2},\ldots ,S_{1,m+N}\\ \ldots \\ S_{k,m+1},S_{1,m+2},\ldots ,S_{1,m+N} \end{array}\right]\left[\begin{array}{c} X_{m+1,1}\\ X_{m+2,2}\\ \ldots \\ X_{m+N,N} \end{array}\right],$$

Such a matrix multiplication can be expressed as:

(3) $$d_{m}=S_{m}x_{m}^{\prime }$$

where $d_{m}={\left[D_{m,1},\ldots ....D_{m,K}\;\right]}^{T}$ consists of the measurements of the m-th pixel across all *K*-times exposures. $S_{m}\in R^{K\times M}$ is the measurement matrix and is a submatrix of the coding matrix S. $x_{m}^{\prime }={\left[X_{m+1,1},X_{m+2,2},...,X_{m+N,N}\right]}^{T}$ is a spatially shifted spectral vector in the original spectral data cube X. To reconstruct data cube X, we need to solve each spatially shifted spectral vector $x_{m}^{\prime }$ in Equation (3) and combine them into the spatially shifted data cube $X^{\prime }$. Additionally, the original spectral data X can be obtained by spatially shifting $X^{\prime }$. To compute $x_{m}^{\prime }$ quickly by matrix inversion in Equation (3) for real-time reconstruction and video acquisition, we need to design the coding matrix S such that all measurement matrixes $S_{m}$ are full-rank.

In the noise-free case, a more direct method is to use the identity matrix as the measurement matrix—i.e., $S_{m}=I_{N}$, where $I_{N}\in R^{N\times N}$ is the identity matrix. Considering $S_{m}$ as a submatrix of the encoding matrix S, when $S_{m}$ is the unit matrix, the imaging process is shown in Figure 2b with the coded slit as a uniform slit. Uniform slits enable parallel measurements in contrast to the single-slit scanning-based spectrometer shown in Figure 2a. Therefore, the detector array under uniform slits receives signals from multiple slit dispersions at the same time, which increases the efficiency of data acquisition by M/K and thus greatly improves the imaging speed. However, in high-speed video imaging with a short integration time and thus a weak signal, noise can cause the dramatic degradation of imaging quality. In Section 2.2, we analyze the noise of the system and propose the use of an S-matrix coded slit rather than a uniform slit. The S-matrix coding slit can suppress the readout noise in the system and greatly improve the imaging quality to achieve real-time spectral video acquisition.

### 2.2. Noise Analysis

We add a noise term to Equation (3):

(4) $$d_{m}=S_{m}x_{m}^{\prime }+e,$$

where $d_{m}\in R^{K}$ consists of measurements of the mth pixel across all K-times exposure, $S_{m}\in R^{K\times M}$ is the measurement matrix, and $x_{m}^{\prime }\in R^{N}$ is the spatially shifted spectral vector. We assume that the elements in the 3D spectral data cube are statistically independent and have the same statistical light intensity $E\left\{x_{m}^{\prime }\right\}=P$. Additionally, we also design the coding matrix as a periodic circular matrix such that the sum of each row of the measurement matrix is the same value, L. Therefore, the statistical light intensity arriving at the detector is LP. $e\in R^{K}$ is the noise, the variance of which is denoted as σ. The noise in our system consists of the photon noise $e_{p}$ and the readout noise $e_{r}$. Photon noise is caused by the random fluctuation in the numbers of photons arriving at the detector at different levels of exposure. The photon noise obeys a Poisson distribution and the variance $\sigma_{p}^{2}$ is related to the light intensity arriving at the detector, $\sigma_{p}^{2}={\left(LP\right)}^{2}$. The readout noise mainly stems from the readout circuits in detectors, which is usually a fixed parameter, and the variance is denoted as $\sigma_{r}^{2}$.

The estimation of the spectral vector, denoted as ${\hat{x}}_{m}^{\prime }$, is obtained by matrix inversion. Then, we define the reconstruction error as:

(5) $$ℰ={\left\|{\hat{x}}_{m}^{\prime }-x_{m}^{\prime }\right\|}^{2}={\left\|S_{m}^{-1}d_{m}-x_{m}^{\prime }\right\|}^{2},$$

The reconstruction error is calculated as [20]:

(6) $$ℰ=Tr\left({\left(S_{m}^{T}S_{m}\right)}^{-1}\right)\sqrt{\frac{\left(\sigma_{p}^{2}+\sigma_{r}^{2}\right)}{N}},$$

By substituting the variance of the photon noise $\sigma_{p}^{2}$ as the intensity of light arriving at the detector, we can obtain:

(7) $$ℰ=Tr\left({\left(S_{m}^{T}S_{m}\right)}^{-1}\right)\sqrt{\frac{\left(L^{2}P^{2}+\sigma_{r}^{2}\right)}{N}},$$

We denote the reconstruction error of the identity measurement matrix as E_I and define the performance gain of a certain measurement matrix $S_{m}$ relative to the identity measurement matrix as:

(8) $$G=\frac{{ℰ}_{I}}{ℰ}=\frac{N}{Tr\left({\left(S_{m}^{T}S_{m}\right)}^{-1}\right)}\sqrt{\frac{P^{2}+\sigma_{r}^{2}}{L^{2}P^{2}+\sigma_{r}^{2}}}=\frac{N}{Tr\left({\left(S_{m}^{T}S_{m}\right)}^{-1}\right)}\sqrt{\frac{1+Q^{2}}{L^{2}+Q^{2}}},$$

where $Q=\sigma_{r}/P$ is the ratio of the system readout noise to the statistical light intensity, which is also known as the inverse relative light intensity of the system. Equation (8) shows that the performance gain is related to both Q and the measurement matrix $S_{m}$. It has been shown in [20] that when the measurement matrix $S_{m}$ is a Hadamard matrix, the performance gain is the highest—i.e., the relative reconstruction error E is the lowest. In this case, we have L=N and $Tr\left({\left(S_{m}^{T}S_{m}\right)}^{-1}\right)=1$. Additionally, we obtain:

(9) $$G=N\sqrt{\frac{1+Q^{2}}{L^{2}+Q^{2}}}=\sqrt{\frac{1+Q^{2}}{1+{\left(\frac{Q}{N}\right)}^{2}}}>1,$$

Equation (9) shows that the reconstruction error of the Hadamard measurement matrix is always smaller than that of the identity measurement matrix. In addition, the performance gain is also related to the ratio of statistical light intensity to readout noise in the system. In our high-speed spectral video imaging system, the light intensity is weak and Q is large, meaning the performance gain achieved from using the Hadamard measurement matrix is large. However, there are difficulties in implementing the Hadamard measurement matrix in a real optical system owing to the ‘−1’ elements in the Hadamard matrix [21]. Therefore, we approximate the Hadamard matrix using an S-matrix with only elements “1” and “0”. First, we use N elements of the m-sequence as the first row of the S-matrix and construct the whole S-matrix using circular shifts. After designing the measurement matrix, we can implement our proposed S-matrix coded slits and build a practical system to validate real-time hyperspectral video acquisition.

In addition to the photon noise and readout noise, the misalignment between the detector and coded slits controlled by displacement platform also leads to noise and reconstruction error. Since the displacement of the coded slits is consistent across the vertical axis, the reconstruction error from this misalignment will cause stripes in the vertical direction. We will show that such a reconstruction error can be compensated for by a calibration process discussed in Section 3.2.

## 3. System Design and Calibration

**Figure 3:**
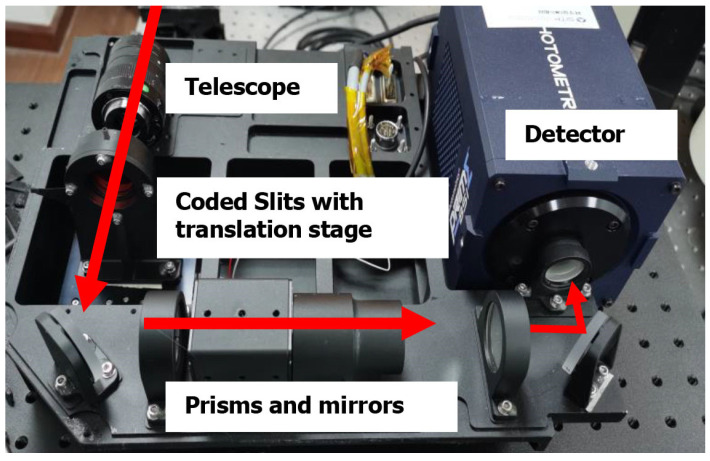
Prototype system for RHVS video acquisition.

To further validate the feasibility of our proposed video acquisition system, we designed and developed a practical prototype. Figure 3 demonstrates the RHVS video acquisition prototype system we developed. The prototype includes a telescope, coded slits with a translation stage, prisms and mirrors, and a detector. The coded slits are placed on the primary focal plane of the telescope to modulate the incident light. The electronically controlled displacement platform is attached to the coded slits to encode in a frame-wise fashion. The coded incident light is then dispersed by a spectrometer and reaches the detector. We used a high-resolution Prime95B sCMOS detector, produced by Teledyne Photometrics, with a resolution of 2048 × 2048 pixels, a 6.5 µm × 6.5 µm element size, and a sufficiently high quantum efficiency (70% at 450 nm, 95% at 550 nm, 70% at 700 nm, and 40% at 900 nm). A v-480 linear motor from Physik Instrumente with a bidirectional repeatability of ±0.1 µm was used, and the motor was closed-loop controlled by a C-981 controller (Physik Instrumente). A grating ruler was used for motor position feedback to ensure the positioning accuracy of the motor. To improve the SNR in our actual experiments, we averaged the measurements across a patch of 2 × 2 pixels in our system corresponding to each wavelength. In Section 3.1, we describe the optical design of the spectrometer system. In Section 3.2, we introduce the design method of the code board and show the actual production effect. In Section 3.3, we perform spectral calibration, correct the central wavelength offset, and finally determine the coding matrix of the aliased signal.

### 3.1. Optical Design

The RHVS requires a good dispersion consistency in a wide field of view (FoV), especially for the spectrometer in the system. Therefore, we designed a prism spectroscopic path with a large field of view. The design parameters are shown in Table 1.

**Figure 4:**
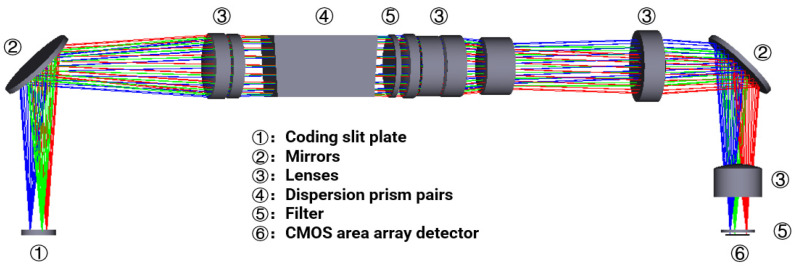
Optical layout of the system.

As shown in Figure 4, the coding slits were placed at the object plane of the spectrometer and at the focal plane of the optical lens. The detector was placed at the focal plane of the spectrometer. We used prism sets rather than grating to disperse the incident light, which does not suffer from higher-order dispersive light and reaches a higher dispersion efficiency. We also applied the symmetrical structure of the prism set to reduce the smiling and keystone effects. Extra lenses were adapted to expand the FoV, as well as to correct aberration. The bandwidth of the bandpass filter was 450 nm–900 nm, which was mainly used to reduce the influence of an optical signal outside the bandwidth on the image effect. We used the modulation transfer function (MTF) to evaluate the imaging quality of the optical design. The Nyquist frequency of the system was computed as 38.46 lp/mm. Under such a frequency, the MTF of the spectrometer at different wavelengths was close to the diffraction limit and larger than 0.65.

### 3.2. Implementation and Calibration

**Figure 5:**
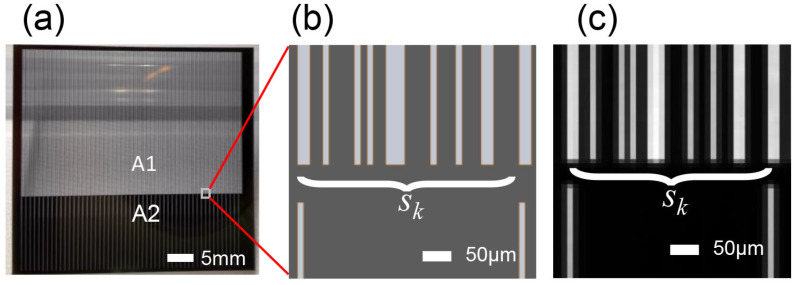
The layout of the slits on the code board. (**a**) A photo of the coding plate, on which a coded slit is in the region A1 and the region A2 contains uniform slits with 0.455 mm intervals between each slit. (**b**) The designed coding slits. (**c**) Part of an image on the detector corresponding to (**b**) at 543 (±10) nm monochromatic light.

Most coded aperture spectral imaging systems use a digital micromirror device (DMD) [22], liquid crystal tunable filters (LCTF) [23], or a coding plate to achieve spectral coding. The small gaps between the micromirrors in a DMD lead to signal distortion owing to diffraction, especially at the infrared band. LCTFs have limited light throughput and thus degrade the performance. As a result, we used a coding plate as the coding device in our proposed system. The coding plate is made of translucent quartz glass as the substrate, the quartz glass is coated with an opaque chromium layer, and the translucent slit is etched on the chromium layer by a photolithography process to serve as the “0” and “1” codes for “transmitting” and “occluding”, respectively. The pattern of the coding plate consists of the coded slit area A1 and the uniform slit area A2. A1 is used to verify the imaging effect of the coded slits, and the slit position is determined using the m sequence as the first-row element of the S matrix, with the whole coding plate being spread periodically. A2 is used for the calibration of the system parameters, and a single slit is placed at fixed intervals to form a uniformly distributed array of slits. The structure of the coding plate is shown in Figure 5. Figure 5a shows the physical diagram of the coding plate, and Figure 5b gives the slit arrangement of the coding slit and the uniform slit. The dispersion width of the spectrometer assembly was 35 pixels wide (including 30 effective bands), and the spacing of each slit in A2 was 35 pixels to ensure that the spectral signals at the focal plane of the detector did not overlap. Each slit in A1 was 120 mm long, and the unit width was 13 um. The slit length in A2 was 20 mm and the width was 13 um. To verify whether the manufactured coding plate was suitable for our proposed system, we fed monochromatic light into the system and subsequently captured the image of the coding plate. Figure 5c shows the image we captured in our real prototype, which is consistent with Figure 5b.

### 3.3. Calibration and Correction

In this section, the correction parameters are first obtained by spectral alignment in order to obtain stable measurements; then, spectral calibration is performed by spectral sweeping. Finally, the center of the calibration is swept again to determine the coding elements of each coding region for data reconstruction.

#### 3.3.1. Correction of Central Wavelength

**Figure 6:**
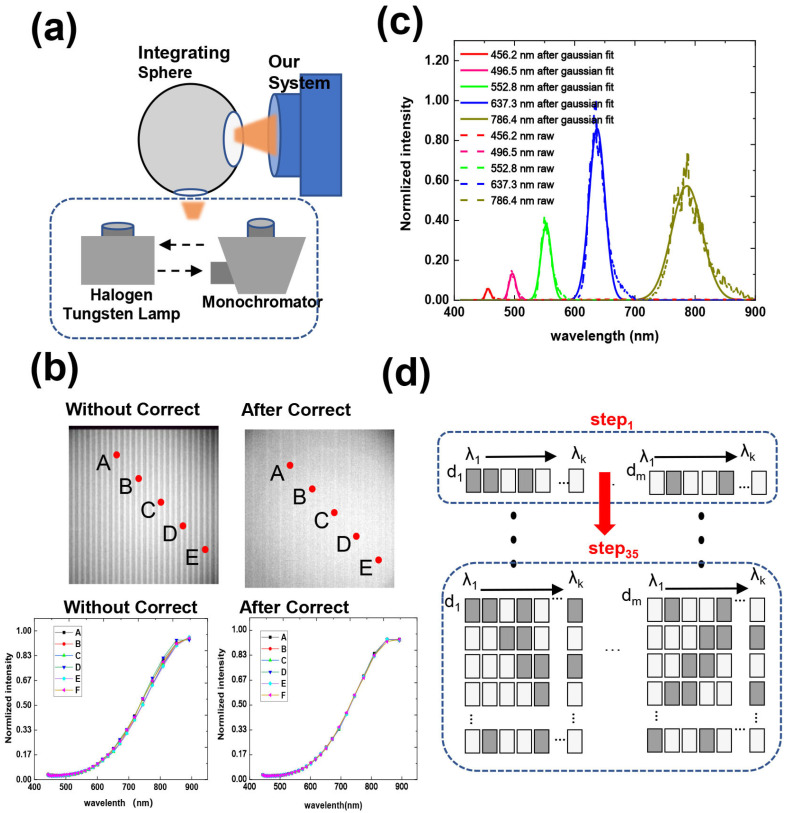
Process and results of system calibration. (**a**) Overview of our methods and design for calibration. (**b**) Comparisons between images and spectrum with and without spectral correction. (**c**) Fitting results of measured spectral response. (**d**) Procedure for acquiring coding matrix. Throughout the entire process, the motor steps 35 times, and each time, it observes the response of the detector in 35 bands to determine the coding values in all states.

The consistency of the measurement is degraded by the movement error of the slit and the dispersion rate difference of the spectroscopic component, which causes central wavelength bias. We eliminated this error by spectral alignment and a calibration process similar to the correction of the non-uniformity of the detector. The experimental setup is shown in Figure 6a. The incident light from the tungsten halogen lamp is fed into the integrating sphere to produce a uniform surface light source with a whole spectral region. A measurement from the integrating sphere is collected using the uniform slit region A2, while the deviation of the spectral curve at each point of the detector from the standard curve is calculated by curve fitting to form a correction matrix. Then, the response curve is calculated by the correction matrix to correct the deviation. The specific experimental process is as follows: First, we used a halogen lamp and integrating sphere to provide uniform light to the system. Then, we moved the coding plate to obtain the spectral three-dimensional cube of the integrating sphere. The mean spectral values of all spatial points in the data were selected as the standard spectrum, and the deviation between the central wavelength of each spatial point and the standard spectrum was calculated using the cubic spline difference method. This process can be better illustrated by an example: let the resulting standard spectrum be y (y is a 30-band curve), with the central wavelength denoted as x. The spectrum measured at a point in space is denoted as $y^{\prime }$, and the actual central wavelength $x^{\prime }$ corresponding to $y^{\prime }\;$ is calculated by the difference as follows:

(10) $$x^{\prime }=fit\left(y,x,y^{\prime }\right),$$

Then, the actual $x^{\prime }$ value corresponding to $y^{\prime }$ is obtained, and the actual response value yy corresponding to x can be obtained by using this method again:

(11) $$yy=fit\left(x^{\prime },y^{\prime },x\right),$$

Figure 6b shows the single-band imaging results of the integrating sphere before and after correction. Since the deviation occurs only in the spectral direction and remains consistent in the column direction, the actual results show obvious streaks in the single-band image, while the corrected spectral curve has a consistent response in each band and the streaks are significantly weakened.

#### 3.3.2. Spectral Calibration

The halogen tungsten lamp in Figure 6a was switched to a monochromator, and we used the same system for the uniform slits. The spectral calibration was performed using the sweep method to conduct Gaussian fitting to obtain the response curve of each pixel. Then, we calculated the central wavelength of the Gaussian curve, which is denoted as $\lambda_{1},\lambda_{2},..,\lambda_{35}$ (the useful band is $\lambda_{1},\lambda_{2},..,\lambda_{30}$). The calibration results are shown in Figure 6c. The original data of the spectral calibration and the results of the Gaussian fitting are shown in Figure 6c.

#### 3.3.3. Calibration of Coded Values

After dispersion, the space point in any FoV reaches the detector at a fixed position. The light intensity received by the detector is the accumulation of light intensity after the dispersion of multiple adjacent points in the FoV, and the bands of these signals must be different. Whether the light from a space point can enter the system depends on whether the point has a slit at the corresponding position of the code board. We exploited the characteristic of one-to-one correspondence between the mixed signal bands on the pixel and the FoV to determine the corresponding coding value of the pixel using single-band scanning. When the coding board is fixed at a certain position, monochromatic light with a center wavelength, as shown in Figure 6c, is used as the input to determine the coding situation of this group of spatial points. Then, by moving the coding board 35 times, a coding matrix of 35 × 35 can be obtained on each pixel.

The experimental process is shown in Figure 6d. The corresponding values of the pixels on the detector were recorded at step 1 to obtain the first row of the encoding matrix for each pixel. We then acquired the complete coding matrix for each pixel by translating the coded slits by repeating step 1 to step 35.

## 4. Results

In this section, we verify the imaging capability of the system by testing uniform slits and coded slits. The test is divided into two parts: SNR verification and video spectral imaging verification. In this section, we demonstrate the imaging advantages of coded slits using the imaging results from both inside and outside the laboratory, and the video spectral imaging capability is verified.

**Figure 7:**
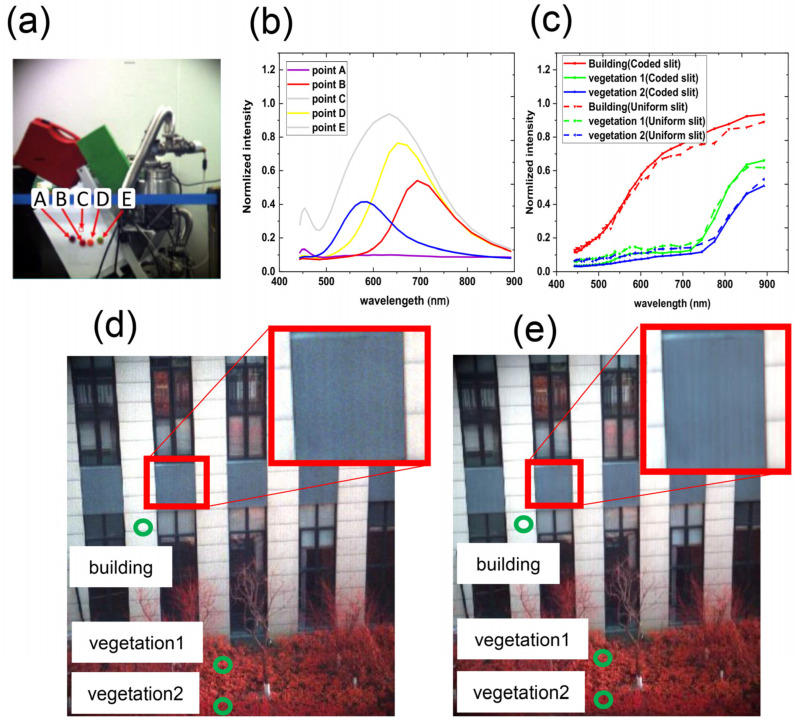
Imaging results of our prototype. (**a**) RGB photo of an indoor scene. (**b**) The spectrum of chosen pixels in (**a**) captured by our prototype with uniform slits. (**c**) Spectrum of building and vegetation captured by our prototype with coded slits and uniform slits. (**d**,**e**) Synthetic pseudo-color images from the spectral image captured by the protype with uniform slits and coded slits, respectively. The magnified area captured by the prototype with coded slits is much less noisy than that of the uniform slits, which shows the considerable denoising effects of the coded slits.

We first used uniform slits in our prototype to validate the system design. Figure 7a shows an RGB image of an indoor scene. The photo was synthesized by spectral images at 638 nm, 522 nm, and 463 nm that were captured by our prototype with uniform slits. Figure 7b shows the spectrum of chosen pixels in Figure 7a. The indoor scene was illuminated by an LED, which has nearly uniform radiance distribution at approximately 400 nm–800 nm; however, it is weak at 800 nm and above. Figure 7d,e shows the false-color photos of an outdoor scene captured by the prototypes with uniform slits and coded slits, respectively. The photos were synthesized by spectral images at 894 nm, 522 nm, and 463 nm. The magnified area in the red frame captured by the prototype with coded slits is significantly less noisy than that with uniform slits, which demonstrates the noise suppression of the coded slits. Figure 7c shows the spectrum of three typical targets in Figure 7d,e. The spectra captured by the prototypes with uniform slits and coded slits were consistent.

**Figure 8:**
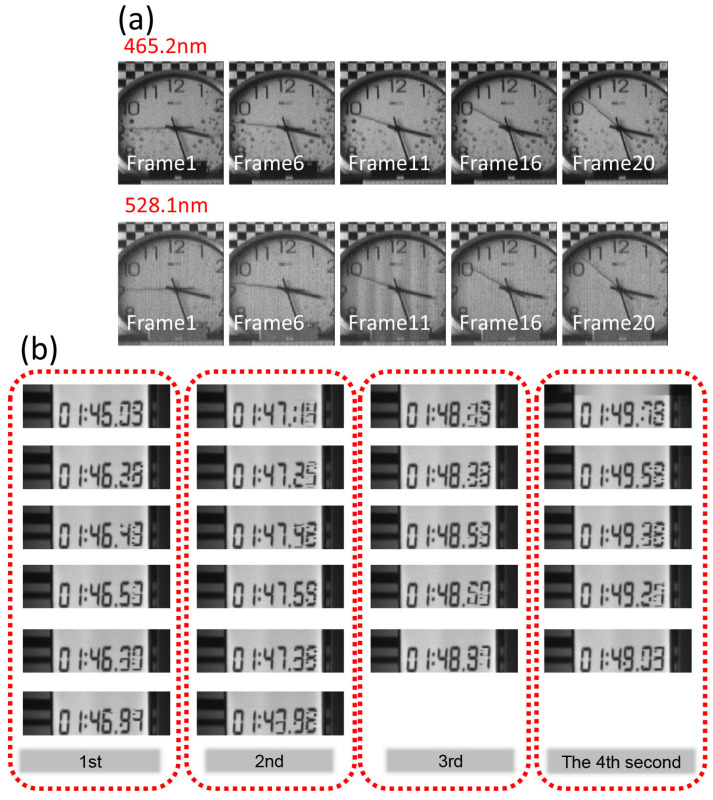
Spectral video results. (**a**) Imaging results for the clocks, including two wavebands with central wavelengths of 465.2 nm and 528.1 nm. (**b**) Imaging results for the timers with a total length of 4 s and 5 or 6 frames per second. We chose band 16 (central wavelength: 522 nm) for display.

The imaging speed of the data cube was determined by the acquisition speed and the reconstruction speed. It took 0.14 s for our prototype to obtain a data cube (the frame rate was 250 Hz, and one data cube was obtained every 35 frames), and the average time taken to rebuild a data cube was 0.05 s/cube. The calculated imaging speed of the system was 5.26 cube/s (Win10, Matlab2016, processor frequency 2.4 GHz). The reconstruction process largely involves operations, and thus the time for reconstruction will be significantly reduced if parallel processing is used. Clocks and timers were used as imaging targets to verify the video capture capabilities of the RHVS. As shown in Figure 8a, the second hand of a clock was regarded as a continuously moving target. It was shown that the imaging speed was sufficient in capturing fast-moving targets. Since the clock contains different colors, we present two images with central wavelengths of 465.2 and 528.1 nm to show the differences.

In addition, a timer was further used as a test target. The timer information includes minutes, seconds, and milliseconds. We captured 4 s in a row, and each second of data contained 5–6 frames of the image cubes. As shown in Figure 8b, the minute and second number of the timer were well captured; however, the millisecond numbers were blurred due to the frame rate used (5 Hz). Compared with CASSI, the most representative computational imaging spectrometer, and ORRIS, a direct imaging spectrometer, whose imaging times are usually 1000 and 4 s, respectively, our system shows a speed advantage.

## 5. Conclusions

In this paper, a video hyperspectral imaging method based on coded slits is proposed, and a prototype is successfully developed. The prototype can capture 30 spectral channels in the spectral region of 450–900 nm with a spatial resolution of 635 × 525. A coded slit full sampling video spectral imaging system that can be used for real-time imaging through parsing is proposed; this allows the data reconstruction time to be reduced to the millisecond level and enables a spectral imaging speed of more than 5 cube/s.

Compared to other computational spectral imaging solutions that sacrifice certain dimensions for higher imaging speed, our proposed RHVS balances the spatial, spectral, and temporal resolution. The reconstruction problem in our RHVS is theoretically well posed, allowing for fast reconstruction via a close-form solution. In addition, the coded slits increase the light throughput of the RHVS system and achieve a higher imaging speed.

Our system still has some limitations. First, it involves dynamic range limitations. The coded slits allow for a larger amount of light to enter the system, and the increased luminous flux prevents its application in scenes with extremely high light intensities. Second, the performance gain of our RHVS will be low when the photon noise is much larger than the readout noise, i.e., when the incident light is high.

## Figures and Tables

**Table 1:** Detailed optical parameters.

Parameters	Spectrometer Components
Spectral range	0.45–0.9 μm
Spectrometer magnification	×1
Object size	13 mm × 13 mm
Spectroscopic element	Prism pairs
Spectral sampling	15 nm
RMS radius of spot	≤6 μm
Smile	˂3.1 μm
Keystone	˂4.2 μm
MTF (@38.46 l p/mm)	˃0.68@0.45 μm; ˃0.73@0.65 μm; ˃0.69@0.9 μm

## Data Availability

The data presented in this study are available on request from the corresponding author.
